# 4-(4-Pyrid­yl)pyridinium penta­aqua(pyridazine-4,5-dicarboxyl­ato)praseodymate(III)

**DOI:** 10.1107/S1600536810033477

**Published:** 2010-09-25

**Authors:** Zhen-Qin Zhang, Xiao-Dong Xue, Bi-Xia Yao, Xing Ji, Hui-Jun Jiang

**Affiliations:** a School of Pharmacy, Nanjing Medical University, Nanjing, People’s Republic of China; b The Scientific and Technological Information Research Department of Jiangsu, Nanjing, People’s Republic of China

## Abstract

In the title complex, (C_10_H_9_N_2_)[Pr(C_6_H_2_N_2_O_4_)_2_(H_2_O)_5_], the Pr atom is nine-coordinated by nine O atoms from two pyridazine-4,5-dicarboxyl­ate anions and five water mol­ecules. It is noteworthy that there is a protonated bipyridine mol­ecule in the structure. Inter­molecular O—H⋯O, O—H⋯N and N—H⋯N hydrogen bonds are present, resulting in a three-dimensional network.

## Related literature

For general background to metal carboxyl­ate coordination compounds, see: Escuer *et al.* (1997 ▶). For pyridazine dicarb­oxy­lic metal complexes, see: Gryz *et al.* (2006 ▶). For bond-length data, see: Allen *et al.* (1987 ▶). 
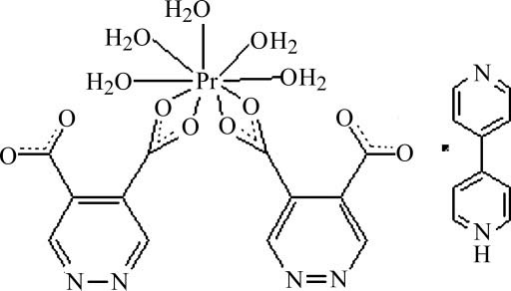


## Experimental

### 

#### Crystal data

(C_10_H_9_N_2_)[Pr(C_6_H_2_N_2_O_4_)_2_(H_2_O)_5_]
*M* *_r_* = 720.37
Orthorhombic, 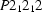

*a* = 11.2726 (17) Å
*b* = 12.0023 (18) Å
*c* = 9.5266 (14) Å
*V* = 1288.9 (3) Å^3^
*Z* = 2
Mo *K*α radiation
μ = 1.97 mm^−1^
*T* = 293 K
0.40 × 0.30 × 0.22 mm

#### Data collection

Rigaku Mercury diffractometer
Absorption correction: multi-scan (*REQAB*; Jacobson, 1998 ▶) *T*_min_ = 0.454, *T*_max_ = 0.649
12497 measured reflections
2358 independent reflections
2280 reflections with *I* > 2σ(*I*)
*R*_int_ = 0.030

#### Refinement

*R*[*F* ^2^ > 2σ(*F* ^2^)] = 0.023
*wR*(*F* ^2^) = 0.052
*S* = 1.09
2358 reflections
216 parameters
6 restraints
H atoms treated by a mixture of independent and constrained refinement
Δρ_max_ = 1.25 e Å^−3^
Δρ_min_ = −0.43 e Å^−3^
Absolute structure: Flack (1983 ▶), 981 Friedel pairs
Flack parameter: −0.014 (18)

### 

Data collection: *CrystalClear* (Rigaku, 1999 ▶); cell refinement: *CrystalClear*; data reduction: *CrystalStructure* (Rigaku, 1999 ▶); program(s) used to solve structure: *SHELXS97* (Sheldrick, 2008 ▶); program(s) used to refine structure: *SHELXL97* (Sheldrick, 2008 ▶); molecular graphics: *ORTEPIII* (Burnett & Johnson, 1996 ▶); software used to prepare material for publication: *CrystalStructure*.

## Supplementary Material

Crystal structure: contains datablocks I, global. DOI: 10.1107/S1600536810033477/bq2230sup1.cif

Structure factors: contains datablocks I. DOI: 10.1107/S1600536810033477/bq2230Isup2.hkl

Additional supplementary materials:  crystallographic information; 3D view; checkCIF report

## Figures and Tables

**Table 1:** Hydrogen-bond geometry (Å, °)

*D*—H⋯*A*	*D*—H	H⋯*A*	*D*⋯*A*	*D*—H⋯*A*
O7—H7*A*⋯O4^i^	0.82 (4)	1.85 (4)	2.662 (3)	171 (5)
O6—H6*B*⋯O3^i^	0.82 (5)	1.93 (5)	2.749 (4)	178 (7)
O6—H6*A*⋯N1^ii^	0.82 (7)	2.07 (6)	2.881 (5)	172 (8)
O5—H5*B*⋯N2^iii^	0.82 (3)	2.14 (4)	2.953 (4)	172 (6)
O5—H5*A*⋯O3	0.82 (4)	2.00 (4)	2.809 (4)	173 (5)
N3—H3*A*⋯N4^iv^	0.91 (1)	1.65 (1)	2.555 (6)	180

Symmetry codes: (i) 
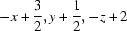
; (ii) 
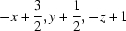
; (iii) 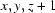; (iv) 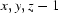.

**Fig. 1.**
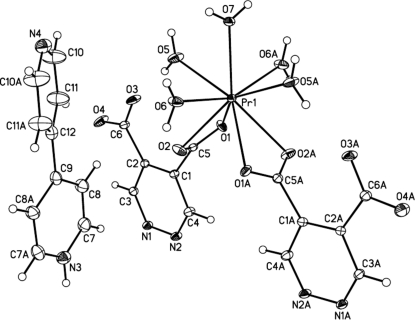
Crystal structure and atom numbering of the title compound, shown with 20% probability displacement ellipsoids. Symmetry code for atoms labelled with A: 1-x, 1-y, z.

**Fig. 2.**
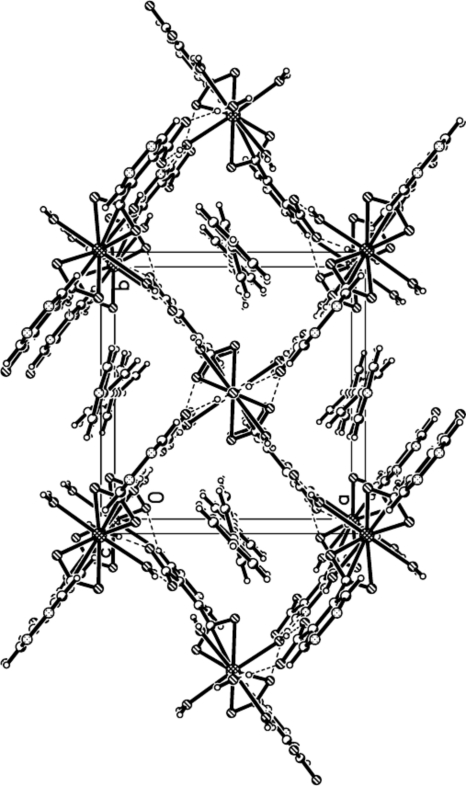
The packing digram of the title compound.

## supplementary crystallographic information

### Comment

In the past few years, investigations on metal carboxylate coordination compounds have become of increasing interest (Escuer *et al.*(1997); Gryz *et al.* 2006). As part of our ongoing investigations in this field we report here the crystal structure of the title compound. In the crystal structure of (I) the Pr atom is coordinated by five oxygen atoms of five water molecules and four oxygen atoms from two pyridazine-4,5-dicarboxylate anions within a distorted orthorhombic coordination symmetry (Figure 1). The bond lengths (Allen *et al.*, 1987) and angles are within normal ranges. The crystal structure contain additional bipyridine molecule that is linked to the complexes *via* O—H···N hydrogen bonding (Figure 2). The complexes are additionally connected by intermolecular O—H···O hydrogen bonding between the carboxyl O atoms and the water H atoms (Table 1 and Figure 2).

### Experimental

A mixture of pyridazine-4,5-dicarboxylic acid (84 mg, 0.5 mmol), NaOH (40 mg, 1.0 mmol), PrCl_3_.6H_2_O (177.7 mg, 0.5 mmol) and 4,4'-bipyridine (78 mg, 0.5 mmol) in warer (10 ml) was placed in a Teflon-lined stainless steel Parr bomb. The bomb was heated at 433 K for 4 d. The bomb was cooled naturally to room temperature, and yellow block crystals of (I) were obtained after several days. Analysis calculated for C_22_H_23_N_6_O_13_Pr: C 36.68, H 3.22, N 11.67%; found: C 36.64, H 3.30, N 11.62%.

### Refinement

Carbon and nitrogen bound H atoms were placed at calculated positions and were treated as riding on the parent C or N atoms with C–H = 0.93 Å, N—H =0.905 Å, and with *U*_iso_(H) = 1.2 *U*_eq_(C, N).The H atoms of water molecules were located in difference Fouier maps, their bond lengths were set to 0.82 Å and afterwards they were refined using a riding model.

### Crystal data

**Table:**

(C_10_H_9_N_2_)[Pr(C_6_H_2_N_2_O_4_)_2_(H_2_O)_5_]	*F*(000) = 720
*M**_r_* = 720.37	*D*_x_ = 1.856 Mg m^−^^3^
Orthorhombic, *P*2_1_2_1_2	Mo *K*α radiation, λ = 0.71070 Å
Hall symbol: P 2 2ab	Cell parameters from 5385 reflections
*a* = 11.2726 (17) Å	θ = 3.3–25.3°
*b* = 12.0023 (18) Å	µ = 1.97 mm^−^^1^
*c* = 9.5266 (14) Å	*T* = 293 K
*V* = 1288.9 (3) Å^3^	Block, yellow
*Z* = 2	0.40 × 0.30 × 0.22 mm

### Data collection

**Table:**

Rigaku Mercury diffractometer	2358 independent reflections
Radiation source: fine-focus sealed tube	2280 reflections with *I* > 2σ(*I*)
graphite	*R*_int_ = 0.030
Detector resolution: 7.31 pixels mm^-1^	θ_max_ = 25.3°, θ_min_ = 3.3°
ω scans	*h* = −13→13
Absorption correction: multi-scan (*REQAB*; Jacobson, 1998)	*k* = −14→13
*T*_min_ = 0.454, *T*_max_ = 0.649	*l* = −11→10
12497 measured reflections	

### Refinement

**Table:**

Refinement on *F*^2^	Secondary atom site location: difference Fourier map
Least-squares matrix: full	Hydrogen site location: inferred from neighbouring sites
*R*[*F*^2^ > 2σ(*F*^2^)] = 0.023	H atoms treated by a mixture of independent and constrained refinement
*wR*(*F*^2^) = 0.052	*w* = 1/[σ^2^(*F*_o_^2^) + (0.0271*P*)^2^ + 0.6051*P*] where *P* = (*F*_o_^2^ + 2*F*_c_^2^)/3
*S* = 1.09	(Δ/σ)_max_ = 0.001
2358 reflections	Δρ_max_ = 1.25 e Å^−^^3^
216 parameters	Δρ_min_ = −0.43 e Å^−^^3^
6 restraints	Absolute structure: Flack (1983), 981 Friedel pairs
Primary atom site location: structure-invariant direct methods	Flack parameter: −0.014 (18)

### Special details

**Table:**

Geometry. All e.s.d.'s (except the e.s.d. in the dihedral angle between two l.s. planes) are estimated using the full covariance matrix. The cell e.s.d.'s are taken into account individually in the estimation of e.s.d.'s in distances, angles and torsion angles; correlations between e.s.d.'s in cell parameters are only used when they are defined by crystal symmetry. An approximate (isotropic) treatment of cell e.s.d.'s is used for estimating e.s.d.'s involving l.s. planes.
Refinement. Refinement of *F*^2^ against ALL reflections. The weighted *R*-factor *wR* and goodness of fit *S* are based on *F*^2^, conventional *R*-factors *R* are based on *F*, with *F* set to zero for negative *F*^2^. The threshold expression of *F*^2^ > σ(*F*^2^) is used only for calculating *R*-factors(gt) *etc*. and is not relevant to the choice of reflections for refinement. *R*-factors based on *F*^2^ are statistically about twice as large as those based on *F*, and *R*- factors based on ALL data will be even larger.

### Fractional atomic coordinates and isotropic or equivalent isotropic displacement parameters (Å^2^)

**Table:**

	*x*	*y*	*z*	*U*_iso_*/*U*_eq_	
Pr1	0.5000	0.5000	0.89791 (2)	0.01750 (8)	
O1	0.4932 (4)	0.32723 (19)	0.7261 (2)	0.0323 (6)	
O2	0.6434 (3)	0.4430 (2)	0.7086 (3)	0.0351 (7)	
O3	0.7353 (3)	0.2019 (3)	0.8030 (3)	0.0471 (8)	
O4	0.8272 (3)	0.0738 (3)	0.6792 (3)	0.0552 (10)	
O5	0.6242 (4)	0.3454 (3)	0.9953 (3)	0.0490 (10)	
H5A	0.651 (4)	0.303 (3)	0.936 (4)	0.049 (15)*	
H5B	0.629 (5)	0.322 (5)	1.076 (2)	0.070 (19)*	
O6	0.6757 (3)	0.6135 (3)	0.9524 (3)	0.0405 (8)	
H6A	0.708 (7)	0.643 (7)	0.885 (5)	0.14 (3)*	
H6B	0.701 (6)	0.640 (5)	1.026 (4)	0.09 (2)*	
O7	0.5000	0.5000	1.1547 (3)	0.0276 (7)	
H7A	0.558 (3)	0.522 (4)	1.198 (4)	0.059 (16)*	
N1	0.7159 (3)	0.1957 (3)	0.3012 (3)	0.0281 (7)	
N2	0.6400 (3)	0.2828 (3)	0.2946 (3)	0.0298 (7)	
N3	1.0000	0.5000	0.3965 (5)	0.0465 (11)	
H3A	1.0000	0.5000	0.3016 (12)	0.042 (14)*	
N4	1.0000	0.5000	1.1284 (5)	0.0510 (12)	
C1	0.6374 (3)	0.2932 (3)	0.5471 (4)	0.0216 (8)	
C2	0.7136 (3)	0.2042 (3)	0.5549 (4)	0.0207 (8)	
C3	0.7492 (3)	0.1586 (3)	0.4262 (4)	0.0258 (8)	
H3B	0.8002	0.0977	0.4287	0.031*	
C4	0.6030 (4)	0.3280 (3)	0.4125 (4)	0.0291 (9)	
H4	0.5502	0.3873	0.4062	0.035*	
C5	0.5883 (3)	0.3579 (3)	0.6721 (4)	0.0233 (8)	
C6	0.7630 (3)	0.1562 (3)	0.6912 (4)	0.0257 (8)	
C7	0.9297 (4)	0.4314 (4)	0.4686 (6)	0.0479 (12)	
H7B	0.8806	0.3830	0.4193	0.058*	
C8	0.9268 (4)	0.4292 (4)	0.6114 (5)	0.0458 (11)	
H8	0.8763	0.3807	0.6584	0.055*	
C9	1.0000	0.5000	0.6851 (6)	0.0367 (11)	
C10	0.9720 (8)	0.4083 (5)	1.0594 (6)	0.081 (3)	
H10	0.9534	0.3437	1.1088	0.097*	
C11	0.9700 (7)	0.4073 (5)	0.9169 (5)	0.073 (2)	
H11	0.9480	0.3427	0.8698	0.087*	
C12	1.0000	0.5000	0.8421 (5)	0.0373 (11)	

### Atomic displacement parameters (Å^2^)

**Table:**

	*U*^11^	*U*^22^	*U*^33^	*U*^12^	*U*^13^	*U*^23^
Pr1	0.02098 (13)	0.01818 (12)	0.01336 (12)	0.0006 (2)	0.000	0.000
O1	0.0269 (14)	0.0402 (13)	0.0298 (12)	−0.001 (2)	0.010 (2)	−0.0098 (10)
O2	0.0478 (18)	0.0265 (13)	0.0310 (16)	−0.0096 (13)	0.0152 (13)	−0.0092 (12)
O3	0.072 (2)	0.0531 (19)	0.0159 (14)	0.0347 (16)	−0.0059 (15)	−0.0025 (14)
O4	0.077 (2)	0.059 (2)	0.0293 (17)	0.0450 (19)	−0.0121 (17)	−0.0020 (16)
O5	0.079 (3)	0.049 (2)	0.0189 (18)	0.0398 (19)	−0.0008 (17)	−0.0046 (16)
O6	0.042 (2)	0.058 (2)	0.0207 (16)	−0.0254 (17)	−0.0008 (15)	0.0003 (16)
O7	0.0284 (18)	0.0378 (18)	0.0167 (15)	−0.003 (4)	0.000	0.000
N1	0.0346 (19)	0.0321 (18)	0.0176 (16)	0.0084 (14)	0.0010 (14)	−0.0026 (14)
N2	0.042 (2)	0.0306 (17)	0.0165 (16)	0.0100 (16)	−0.0005 (15)	−0.0023 (14)
N3	0.053 (3)	0.049 (3)	0.038 (3)	0.007 (6)	0.000	0.000
N4	0.061 (3)	0.051 (3)	0.041 (3)	0.015 (8)	0.000	0.000
C1	0.026 (2)	0.0207 (19)	0.0184 (18)	−0.0002 (15)	−0.0018 (15)	−0.0006 (15)
C2	0.0216 (19)	0.0222 (19)	0.0183 (18)	−0.0001 (15)	−0.0010 (14)	−0.0017 (15)
C3	0.029 (2)	0.027 (2)	0.022 (2)	0.0063 (17)	0.0017 (15)	−0.0021 (16)
C4	0.040 (2)	0.025 (2)	0.022 (2)	0.0088 (17)	0.0001 (17)	−0.0015 (16)
C5	0.027 (2)	0.026 (2)	0.0165 (18)	0.0067 (16)	−0.0017 (15)	0.0037 (16)
C6	0.032 (2)	0.026 (2)	0.0188 (19)	0.0056 (17)	−0.0008 (16)	0.0028 (17)
C7	0.042 (3)	0.043 (3)	0.058 (3)	−0.009 (2)	−0.005 (2)	0.002 (2)
C8	0.045 (3)	0.048 (3)	0.044 (3)	−0.011 (2)	0.000 (2)	−0.003 (2)
C9	0.032 (3)	0.031 (2)	0.048 (3)	−0.008 (7)	0.000	0.000
C10	0.143 (9)	0.050 (3)	0.048 (3)	−0.022 (4)	0.007 (4)	0.003 (2)
C11	0.118 (8)	0.056 (3)	0.044 (3)	−0.028 (4)	0.001 (3)	−0.001 (2)
C12	0.034 (3)	0.033 (3)	0.045 (3)	0.001 (7)	0.000	0.000

### Geometric parameters (Å, °)

**Table:**

Pr1—O7	2.446 (3)	N3—C7^ii^	1.333 (5)
Pr1—O6	2.459 (3)	N3—C7	1.333 (5)
Pr1—O6^i^	2.459 (3)	N3—H3A	0.905 (10)
Pr1—O5	2.503 (3)	N4—C10	1.321 (6)
Pr1—O5^i^	2.503 (3)	N4—C10^ii^	1.321 (6)
Pr1—O2^i^	2.516 (3)	C1—C2	1.373 (5)
Pr1—O2	2.516 (3)	C1—C4	1.403 (5)
Pr1—O1	2.643 (2)	C1—C5	1.525 (5)
Pr1—O1^i^	2.643 (2)	C2—C3	1.401 (5)
Pr1—C5	2.921 (4)	C2—C6	1.526 (5)
Pr1—C5^i^	2.921 (4)	C3—H3B	0.9300
O1—C5	1.245 (5)	C4—H4	0.9300
O2—C5	1.246 (4)	C7—C8	1.361 (7)
O3—C6	1.238 (5)	C7—H7B	0.9300
O4—C6	1.231 (4)	C8—C9	1.377 (5)
O5—H5A	0.82 (4)	C8—H8	0.9300
O5—H5B	0.82 (3)	C9—C8^ii^	1.377 (5)
O6—H6A	0.82 (7)	C9—C12	1.496 (7)
O6—H6B	0.82 (5)	C10—C11	1.357 (7)
O7—H7A	0.82 (4)	C10—H10	0.9300
N1—C3	1.326 (5)	C11—C12	1.363 (6)
N1—N2	1.354 (4)	C11—H11	0.9300
N2—C4	1.315 (5)	C12—C11^ii^	1.363 (6)
			
O7—Pr1—O6	77.81 (8)	C5—O1—Pr1	90.0 (2)
O7—Pr1—O6^i^	77.81 (8)	C5—O2—Pr1	95.9 (2)
O6—Pr1—O6^i^	155.63 (15)	Pr1—O5—H5A	115 (4)
O7—Pr1—O5	68.24 (7)	Pr1—O5—H5B	130 (4)
O6—Pr1—O5	83.23 (14)	H5A—O5—H5B	114 (5)
O6^i^—Pr1—O5	87.78 (16)	Pr1—O6—H6A	115 (6)
O7—Pr1—O5^i^	68.24 (7)	Pr1—O6—H6B	132 (5)
O6—Pr1—O5^i^	87.78 (16)	H6A—O6—H6B	111 (7)
O6^i^—Pr1—O5^i^	83.23 (14)	Pr1—O7—H7A	120 (3)
O5—Pr1—O5^i^	136.47 (14)	C3—N1—N2	118.7 (3)
O7—Pr1—O2^i^	135.77 (7)	C4—N2—N1	118.6 (3)
O6—Pr1—O2^i^	121.20 (10)	C7^ii^—N3—C7	118.0 (6)
O6^i^—Pr1—O2^i^	77.56 (11)	C7^ii^—N3—H3A	121.0 (3)
O5—Pr1—O2^i^	145.74 (10)	C7—N3—H3A	121.0 (3)
O5^i^—Pr1—O2^i^	72.84 (10)	C10—N4—C10^ii^	120.3 (6)
O7—Pr1—O2	135.77 (7)	C2—C1—C4	117.0 (3)
O6—Pr1—O2	77.56 (11)	C2—C1—C5	125.5 (3)
O6^i^—Pr1—O2	121.20 (10)	C4—C1—C5	117.5 (3)
O5—Pr1—O2	72.84 (10)	C1—C2—C3	115.8 (3)
O5^i^—Pr1—O2	145.74 (10)	C1—C2—C6	124.7 (3)
O2^i^—Pr1—O2	88.45 (14)	C3—C2—C6	119.5 (3)
O7—Pr1—O1	128.27 (5)	N1—C3—C2	125.0 (3)
O6—Pr1—O1	126.08 (14)	N1—C3—H3B	117.5
O6^i^—Pr1—O1	70.89 (12)	C2—C3—H3B	117.5
O5—Pr1—O1	70.39 (11)	N2—C4—C1	124.8 (3)
O5^i^—Pr1—O1	142.66 (14)	N2—C4—H4	117.6
O2^i^—Pr1—O1	75.56 (10)	C1—C4—H4	117.6
O2—Pr1—O1	50.33 (10)	O2—C5—O1	123.8 (3)
O7—Pr1—O1^i^	128.27 (5)	O2—C5—C1	117.1 (3)
O6—Pr1—O1^i^	70.89 (12)	O1—C5—C1	119.0 (3)
O6^i^—Pr1—O1^i^	126.08 (14)	O2—C5—Pr1	58.96 (19)
O5—Pr1—O1^i^	142.66 (14)	O1—C5—Pr1	64.81 (18)
O5^i^—Pr1—O1^i^	70.39 (11)	C1—C5—Pr1	174.8 (3)
O2^i^—Pr1—O1^i^	50.33 (10)	O4—C6—O3	125.7 (4)
O2—Pr1—O1^i^	75.56 (10)	O4—C6—C2	116.0 (3)
O1—Pr1—O1^i^	103.45 (10)	O3—C6—C2	118.2 (3)
O7—Pr1—C5	137.44 (7)	N3—C7—C8	122.8 (5)
O6—Pr1—C5	101.81 (12)	N3—C7—H7B	118.6
O6^i^—Pr1—C5	96.11 (11)	C8—C7—H7B	118.6
O5—Pr1—C5	69.47 (9)	C7—C8—C9	118.9 (5)
O5^i^—Pr1—C5	153.75 (10)	C7—C8—H8	120.5
O2^i^—Pr1—C5	81.37 (9)	C9—C8—H8	120.5
O2—Pr1—C5	25.10 (9)	C8—C9—C8^ii^	118.7 (6)
O1—Pr1—C5	25.24 (11)	C8—C9—C12	120.7 (3)
O1^i^—Pr1—C5	89.54 (9)	C8^ii^—C9—C12	120.7 (3)
O7—Pr1—C5^i^	137.44 (7)	N4—C10—C11	120.5 (6)
O6—Pr1—C5^i^	96.11 (11)	N4—C10—H10	119.7
O6^i^—Pr1—C5^i^	101.81 (12)	C11—C10—H10	119.7
O5—Pr1—C5^i^	153.75 (10)	C10—C11—C12	120.8 (5)
O5^i^—Pr1—C5^i^	69.47 (9)	C10—C11—H11	119.6
O2^i^—Pr1—C5^i^	25.10 (9)	C12—C11—H11	119.6
O2—Pr1—C5^i^	81.37 (9)	C11—C12—C11^ii^	117.0 (6)
O1—Pr1—C5^i^	89.54 (9)	C11—C12—C9	121.5 (3)
O1^i^—Pr1—C5^i^	25.24 (11)	C11^ii^—C12—C9	121.5 (3)
C5—Pr1—C5^i^	85.11 (13)		
			
O7—Pr1—O1—C5	−121.79 (19)	C4—C1—C5—O1	90.2 (4)
O6—Pr1—O1—C5	−17.8 (3)	O7—Pr1—C5—O2	−99.9 (2)
O6^i^—Pr1—O1—C5	−177.9 (3)	O6—Pr1—C5—O2	−15.2 (2)
O5—Pr1—O1—C5	−83.3 (2)	O6^i^—Pr1—C5—O2	−178.6 (2)
O5^i^—Pr1—O1—C5	133.4 (3)	O5—Pr1—C5—O2	−93.2 (3)
O2^i^—Pr1—O1—C5	100.5 (2)	O5^i^—Pr1—C5—O2	94.4 (4)
O2—Pr1—O1—C5	0.3 (2)	O2^i^—Pr1—C5—O2	105.1 (2)
O1^i^—Pr1—O1—C5	58.21 (19)	O1—Pr1—C5—O2	179.4 (4)
C5^i^—Pr1—O1—C5	79.5 (3)	O1^i^—Pr1—C5—O2	55.2 (2)
O7—Pr1—O2—C5	107.2 (2)	C5^i^—Pr1—C5—O2	80.1 (2)
O6—Pr1—O2—C5	164.8 (2)	O7—Pr1—C5—O1	80.6 (2)
O6^i^—Pr1—O2—C5	1.7 (3)	O6—Pr1—C5—O1	165.4 (2)
O5—Pr1—O2—C5	78.1 (2)	O6^i^—Pr1—C5—O1	2.0 (2)
O5^i^—Pr1—O2—C5	−128.4 (3)	O5—Pr1—C5—O1	87.3 (2)
O2^i^—Pr1—O2—C5	−72.8 (2)	O5^i^—Pr1—C5—O1	−85.1 (4)
O1—Pr1—O2—C5	−0.3 (2)	O2^i^—Pr1—C5—O1	−74.4 (2)
O1^i^—Pr1—O2—C5	−122.0 (2)	O2—Pr1—C5—O1	−179.4 (4)
C5^i^—Pr1—O2—C5	−97.0 (2)	O1^i^—Pr1—C5—O1	−124.24 (19)
C3—N1—N2—C4	0.6 (5)	C5^i^—Pr1—C5—O1	−99.4 (2)
C4—C1—C2—C3	0.5 (5)	C1—C2—C6—O4	177.7 (4)
C5—C1—C2—C3	−178.8 (3)	C3—C2—C6—O4	−4.3 (6)
C4—C1—C2—C6	178.6 (4)	C1—C2—C6—O3	−1.9 (6)
C5—C1—C2—C6	−0.8 (6)	C3—C2—C6—O3	176.0 (4)
N2—N1—C3—C2	−1.3 (6)	C7^ii^—N3—C7—C8	0.3 (4)
C1—C2—C3—N1	0.7 (6)	N3—C7—C8—C9	−0.5 (7)
C6—C2—C3—N1	−177.5 (4)	C7—C8—C9—C8^ii^	0.2 (4)
N1—N2—C4—C1	0.7 (6)	C7—C8—C9—C12	−179.8 (4)
C2—C1—C4—N2	−1.2 (6)	C10^ii^—N4—C10—C11	−0.9 (6)
C5—C1—C4—N2	178.2 (4)	N4—C10—C11—C12	1.8 (13)
Pr1—O2—C5—O1	0.6 (4)	C10—C11—C12—C11^ii^	−0.9 (6)
Pr1—O2—C5—C1	176.2 (3)	C10—C11—C12—C9	179.1 (6)
Pr1—O1—C5—O2	−0.6 (4)	C8—C9—C12—C11	27.2 (4)
Pr1—O1—C5—C1	−176.1 (3)	C8^ii^—C9—C12—C11	−152.8 (4)
C2—C1—C5—O2	93.8 (5)	C8—C9—C12—C11^ii^	−152.8 (4)
C4—C1—C5—O2	−85.6 (4)	C8^ii^—C9—C12—C11^ii^	27.2 (4)
C2—C1—C5—O1	−90.4 (5)		

Symmetry codes: (i) −*x*+1, −*y*+1, *z*; (ii) −*x*+2, −*y*+1, *z*.

### Hydrogen-bond geometry (Å, °)

**Table:**

*D*—H···*A*	*D*—H	H···*A*	*D*···*A*	*D*—H···*A*
O7—H7A···O4^iii^	0.82 (4)	1.85 (4)	2.662 (3)	171 (5)
O6—H6B···O3^iii^	0.82 (5)	1.93 (5)	2.749 (4)	178 (7)
O6—H6A···N1^iv^	0.82 (7)	2.07 (6)	2.881 (5)	172 (8)
O5—H5B···N2^v^	0.82 (3)	2.14 (4)	2.953 (4)	172 (6)
O5—H5A···O3	0.82 (4)	2.00 (4)	2.809 (4)	173 (5)
N3—H3A···N4^vi^	0.91 (1)	1.65 (1)	2.555 (6)	180

Symmetry codes: (iii) −*x*+3/2, *y*+1/2, −*z*+2; (iv) −*x*+3/2, *y*+1/2, −*z*+1; (v) *x*, *y*, *z*+1; (vi) *x*, *y*, *z*−1.
